# Increased PRP19 in Hepatocyte Impedes B Cell Function to Promote Hepatocarcinogenesis

**DOI:** 10.1002/advs.202407517

**Published:** 2024-10-18

**Authors:** Zhiyong Liu, Xiahui Lin, Danying Zhang, Dezhen Guo, Wenqing Tang, Xiangnan Yu, Feng Zhang, Si Zhang, Ruyi Xue, Xizhong Shen, Ling Dong

**Affiliations:** ^1^ Department of Gastroenterology and Hepatology Shanghai Institute of Liver Disease Zhongshan Hospital Fudan University Shanghai 200030 China; ^2^ Department of Liver Surgery Zhongshan Hospital Fudan University Shanghai 200030 China; ^3^ NHC Key Laboratory of Glycoconjugate Research Department of Biochemistry and Molecular Biology School of Basic Medical Sciences Fudan University Shanghai 200030 China

**Keywords:** hepatocellular carcinoma, immune response, Pre‐mRNA processing factor 19, tumor microenvironment, tumor‐infiltrating B cells

## Abstract

Tumor immune microenvironment is strongly associated with the malignancy behavior of hepatocellular carcinoma (HCC). However, the immune function and regulatory mechanisms of B cells in HCC remain unclear. The expression differences between B cell high‐ and low‐infiltration HCC samples are explored to identify the key regulator. Pre‐mRNA processing factor 19 (PRP19) expression is increased in B cell low‐infiltrated tissues and negatively correlated with the B cell marker, CD20. Inhibition of PRP19 expression promoted B cell infiltration in tumor tissue and impeded HCC growth. Mechanically, the co‐immunoprecipitation (Co‐IP) assay revealed that PRP19 interacts with DEAD‐box helicase 5 (DDX5), leading to ubiquitination and degradation of the DDX5 protein. The attenuated DDX5 impairs CXCL12 mRNA stability to suppress B cell recruitment and plasma cell differentiation via CXCL12/CXCR4 axis. Moreover, the adoptive transfer of CXCR4+ B cells combined with CXCL12 treatment in mice models effectively inhibits HCC development by reshaping the immune response. The expression of PRP19, DDX5, and infiltrating B cells are recognized as clinical prognosis indicators for HCC patients. Overall, this study provides valuable insights into the clinical benefits of HCC immunotherapy by targeting PRP19 and modulating tumor‐infiltrating B cell immune function.

## Graphical abstract

This study investigates the role of B cells in the tumor immune microenvironment of HCC. It identifies PRP19 as a key regulator, with its high expression linked to low B cell infiltration. Inhibiting PRP19 enhances B cell migration and differentiation to reduce HCC growth. The findings suggest that targeting PRP19 may improve HCC immunotherapy.

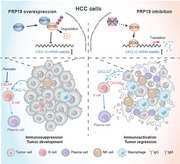

## 1 Introduction

Hepatocellular carcinoma (HCC) is the most common type of liver cancer and remains a health challenge worldwide.^[^ ^1^ ^]^ The majority of HCC cases usually occur in inflamed liver, inflammatory status, and immune responses in the tumor microenvironment (TME), which can markedly affect the biological behavior of malignancy.^[^ ^2^ ^]^ Early investigations on immunotherapy have identified that anti‐tumor responses modulated by immune cells could serve as effective treatment tools for solid or hematological malignancies, especially for T cells and macrophages.^[^ ^3^ ^]^ Tumor‐infiltrating B cells are a critical component of the adaptive immune system in the TME and exhibit remarkable functional diversity with different subsets, such as antigen‐presenting cells (APC), cytokine‐producing cells, and antibody‐secreting plasma cells.^[^ ^4^ ^]^ Further investigation of B cells within the TME may provide important methods for modulation of the immune response and meaningful therapeutic intervention for HCC.

The functional features of tumor‐infiltrating B cells and their effects on tumor immunity are still controversial.^[^ ^5^ ^]^ B cells are highly heterogeneous in human malignant tumors and appear distinct functions of pro‐ and anti‐tumor roles.^[^ ^6^ ^]^ Early studies reported that CD20+ B cells exert anti‐tumor properties;^[^ ^4^ ^]^ however, regulating B cells (Bregs) primarily drive tumor progression by upregulating IL‐10 and TGF‐β.^[^ ^7^ ^]^ In colorectal tumors, researchers have identified a special subset of B cells induced by leucine metabolism, namely LARS B cells, which produce TGF‐β1 by inhibiting mitochondrial NAD + regeneration in B cells and oxidative metabolism to promote tumor immune evasion.^[^ ^8^ ^]^ To date, the key factors that modulate B‐cell infiltration and function in tumor development remain to be elucidated. Pre‐mRNA processing factor 19 (PRP19) is a critical member of the PRP19‐associated complex that exerts multiple biological functions,^[^ ^9^ ^]^ such as DNA damage response,^[^ ^10^ ^]^ tumor radiotherapy resistance,^[^ ^11^ ^]^ and protein ubiquitin.^[^ ^12^ ^]^ However, the relationship between PRP19 expression and B cell function in the TME of HCC is largely unclear.

In this study, we explored the expression features between B cell high and low infiltration levels of HCC samples and identified PRP19 as a key mediator for B cell infiltration. High expression of PRP19 impeded B cell recruitment in clinical HCC patients and suggested poor prognostic outcomes. As the underlying mechanism, we found PRP19 knockdown in HCC cells inhibited DDX5 protein degradation, thus promoting CXCL12 expression to induce B cell recruitment via binding the receptor CXCR4. Collectively, our study revealed the relationship between PRP19 expression in HCC cells and tumor‐infiltrating B cell function and provided novel clinical benefits for HCC immunotherapy.

## 2 Results

### 2.1 PRP19 Expression is Increased in HCC Samples Containing Low Infiltration of B Cells

**Figure 1:**
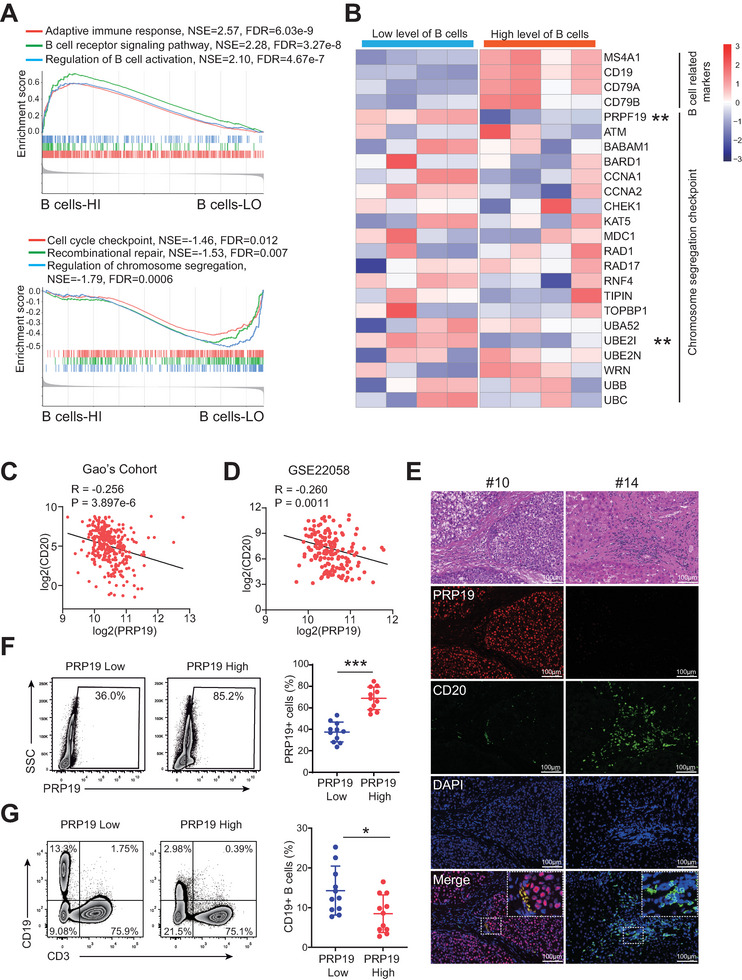
PRP19 expression was increased in B cell high infiltrated HCC samples. A) GSEA analysis of signal pathways in HCC samples with high and low B cell infiltration. B) Expression heatmap of chromosome segregation checkpoints and B cell‐related markers between B cell high and low infiltrated HCC samples. C) Correlation analysis of PRP19 and CD20 in Gao's cohort. D) Correlation analysis of PRP19 and CD20 in GSE22058 dataset. E) Representative IF images of PRP19 and B cells in HCC tissues. F) The HCC samples were divided into two groups according to PRP19 expression level. G) FCM analysis revealed that tumor‐infiltrating B cells were attenuated in PRP19‐high expression HCC patients. * p < 0.05, **p < 0.01, ***p < 0.001 by student's t‐test (B, F, G), Pearson correlation analysis (C, D). Abbreviations: HCC, hepatocellular carcinoma; GSEA, gene set enrichment analysis; HE, hematoxylin eosin; FCM, flow cytometry.

To explore the factors mediating B cell infiltration, we first assessed the infiltration scores of B cell in our HCC samples sequencing cohort^[^ ^13^ ^]^ using ssGSEA by R GSVA package (Figure S1A, Supporting Information). The top four B‐cell high‐infiltrated samples and low‐infiltrated samples were selected to investigate the differential expression genes and signal pathways. GSEA analysis showed that HCC samples with high B cell infiltration levels were mainly associated B cell‐related immune signals, such as “adaptive immune response”, “B cell receptor signaling pathway”, and “regulation of B cell activation” (**Figure** **1**A). In low B cell infiltrated samples, we found that “Cell cycle checkpoint”, “Recombinational repair”, and “regulation of chromosome segregation” had significant enrichment scores (Figure 1A), indicating chromosome segregation‐related pathways might affect B cell infiltration in HCC. The heatmap revealed that B cell‐related markers including MS4A1 (CD20), CD19, CD79A, CD79B, were highly expressed in HCC tissues with high B cell scores (Figure 1B). We further obtained chromosome segregation checkpoints from PathCards (https://pathcards.genecards.org/) and compared their expression levels between B cell high and low infiltration samples. PRP19 and UBE2I displayed relatively low expression levels in high B cell score samples (Figure 1B). By exploring the TCGA‐LIHC dataset, we found that PRP19 and UBE2I expression levels were increased in HCC tissues (Figure S1B, Supporting Information), and PRP19 was negatively correlated with CD20 expression (R = −0.35, p = 7.2e–15, Figure S1C, Supporting Information). No significant correlation was found between UBE2I and CD20 levels in TCGA‐LIHC.

To validate the relationship between PRP19 and CD20, The Zhongshan Hospital HCC dataset (Gao's cohort),^[^ ^14^ ^]^ OEP000321, including 159 patients, was downloaded from the National Omics Data Encyclopedia (NODE). We found that PRP19 was increased and CD20 was downregulated in HCC tissues compared with adjacent HCC tissues (Figure S1D, Supporting Information). Pearson analysis indicated that PRP19 was negatively correlated with CD20 expression in Gao's cohort (R = −0.256, p = 3.897e–6) (Figure 1C). Similar results for PRP19 and CD20 were obtained in another independent HCC sequencing dataset, GSE22058 (Figure 1D; Figure S1E, Supporting Information). We further performed immunofluorescence (IF) in clinical HCC tissues and found that CD20+ B cell infiltration was decreased in PRP19 high‐expressed HCC samples (Figure 1E). Using flow cytometry (FCM), we investigate PRP19 expression and CD3‐CD19+ B cell ratio in 22 clinical HCC tissues. Results showed that PRP19 was mainly expressed in non‐immune cells (CD45‐) when compared with CD45+ immune cells (Figure S1F, Supporting Information). We then divided the 22 HCC samples into PRP19‐high and PRP19‐low groups according to the mean PRP19+ cell proportion (Figure 1F). FCM results showed that B cells (CD3‐CD19+) had a lower proportion of PRP19‐high group HCC tissues when compared with the PRP19‐low group (Figure 1G). Next, we investigated the prognostic values of PRP19 and CD20 in HCC patients. Kaplan–Meier analysis indicated that high expression of PRP19 and low expression of CD20 in HCC patients had poor prognosis in the TCGA‐LIHC dataset (Figure S1G, Supporting Information) and Gao's cohort (Figure S1H, Supporting Information), and patients with high PRP19 and low CD20 levels had the worst prognosis (Figure S1I, Supporting Information). Collectively, these findings suggest that high expression of PRP19 might impede B cell infiltration in HCC tissues, and the levels of PRP19 and B cell marker CD20 are associated with the prognostic outcomes of HCC patients.

### 2.2 PRP19 Deficiency Inhibits HCC Progression Via Promoting B Cell Infiltration

**Figure 2:**
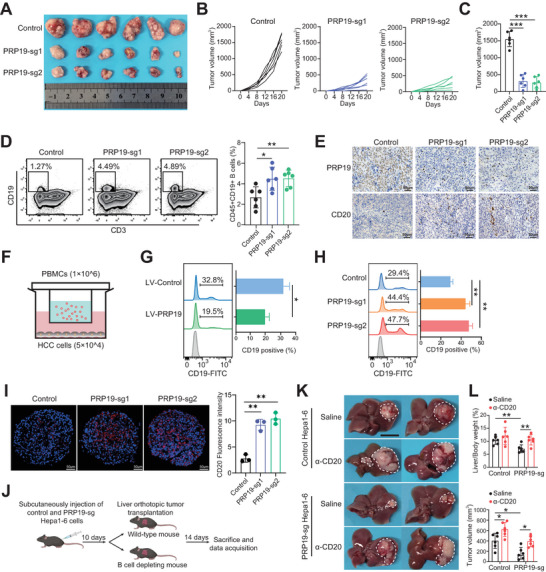
PRP19 deficiency promoted B cell infiltration to impede HCC development. A) Subcutaneous mice tumor models were constructed by control and PRP19 knockout HCC cells. B) The tumor volumes were recorded every four days and growth curves were plotted. C) The tumor volumes were compared at the endpoint. D) CD19+ B cell proportion in mouse tumor tissues was investigated by FCM. E) PRP19 and CD20 expression were measured using IHC in mouse tumor tissues. F) PBMCs were co‐cultured with indicated HCC cells in the transwell system. B cell migration ability was analyzed by FCM when co‐cultured with PRP19 overexpression G) and knockout HCC cells H). I) B cell infiltration level was measured by IF staining in control and PRP19 knockout HCC spheroids. J) Orthotopic liver tumor was constructed in wild‐type and B cell deleting mice. K) The liver images of mouse orthotopic tumors in each group. L) The liver, body weights of the mouse, and tumor volume were measured at the endpoint. *p < 0.05, **p < 0.01, ***p < 0.001 by student's t‐test (C, D, G) and ANOVA test (H, I, K). Abbreviation: PBMC, peripheral blood mononuclear cell.

To gain new insights into PRP19 in B cell functions during HCC pathogenesis, we established PRP19 knockout mouse HCC cells in vitro (Figure S2A, Supporting Information) and subcutaneously injected control and PRP19 knockout HCC cells into mice. Strikingly, we observed smaller tumor sizes and slower tumor growth rates in PRP19 knockout group mice (**Figure** **2**A–C). FCM revealed higher levels of B cell in PRP19 knockout mouse HCC tissues (Figure 2D). CD4+T, CD8+T, and CD115+monocytes showed no significant differences between control and PRP19 knockout groups (Figure S2C,D, Supporting Information). Immunohistochemical staining for PRP19 and CD20 (B‐cell marker) supported the FCM findings (Figure 2E).

To investigate the role of PRP19 in modulating B‐cell migration, we collected peripheral blood mononuclear cells (PBMCs) and co‐cultured them with HCC cells in vitro (Figure 2F). Flow cytometry (FCM) was used to investigate the ratio of recruited CD19+ B cells. The results suggested that B cells had a poor migration tendency when co‐cultured with PRP19 overexpressed HCC cells (Figure 2G; Figure S2B, Supporting Information). Nevertheless, the proportion of recruited B cells was increased in the PRP19 knockdown co‐cultured group (Figure 2H). In addition, we found that alterations in PRP19 expression in HCC cells had no influence on the migration ability of T cells (Figure S2E,F, Supporting Information). B cell migration ability was further measured in 3D HCC spheroids, revealing that there was a higher infiltration of B cells in PRP19 knockdown HCC spheroids (Figure 2I). Next, we explored the role of B cells in the development of HCC. First, we deleted mouse B cells using an anti‐CD20 antibody, and the deletion effect was confirmed by FCM (Figure S2G, Supporting Information). Furthermore, the orthotopic liver tumor model was constructed in B cell deletion and wild‐type mice using control and PRP19 knockout Hepa1‐6 cells (Figure 2J). We found that PRP19 knockout suppressed mouse HCC development compared to that in the control group. However, B cell deletion dampened the anti‐tumor effect of PRP19 knockout in mouse HCC development (Figure 2K,L). These findings suggest that inhibition of PRP19 in HCC cells might induce B cell infiltration to impede hepatocarcinogenesis and that deletion of B cells could impair the tumor‐suppressive role of PRP19 inhibition.

### 2.3 PRP19 Suppresses CXCL12 Expression to Impede B Cell Function

**Figure 3:**
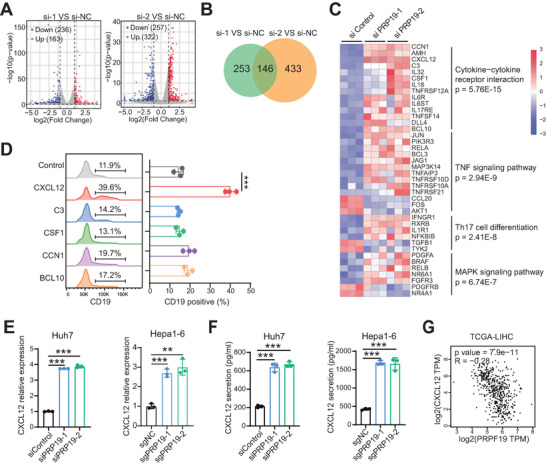
PRP19 suppressed CXCL12 expression in HCC cells. A) The volcano plots of RNA‐sequence in two PRP19 knockdown HCC cells. B) The Venn diagram indicated the co‐differentially expressed genes in two PRP19 knockdown HCC cells. C) The heatmap of key differentially expressed genes in PRP19 knockdown HCC cells. D) B cell migration ability was analyzed by FCM after being treated with CXCL12, C3, CSF1, CCN1, and BCL10. E) CXCL12 expression was analyzed by qPCR in PRP19 knockdown HCC cells. F) CXCL12 protein expression was analyzed by ELISA in PRP19 knockdown HCC cells. G) PRP19 was negatively correlated with CXCL12 mRNA expression in TCGA‐LHC. **p < 0.01, ***p < 0.001 by student's t‐test (D, E, F) and Pearson correlation analysis (G).

We performed RNA sequencing in control and PRP19 knockdown HCC cells to investigate downstream molecular mechanisms. Here, 399 differentially regulated genes, including 163 upregulated and 236 downregulated genes, were identified in the siPRP19‐1 group and 579 differentially regulated genes, including 322 upregulated and 257 downregulated genes, were identified in the siPRP19‐2 group (**Figure** **3**A; Figure S3A, Supporting Information). Comparing the two knockdown groups, we identified 146 co‐differentially regulated genes, including 107 upregulated and 39 downregulated genes (Figure 3B). KEGG pathway enrichment showed that “cytokine−cytokine receptor interaction”, ″pathways in cancer, and “TNF signaling pathway” were the most highly enriched pathways (Figure S3B, Supporting Information). GO enrichment of the co‐differentially regulated genes revealed that the top three enriched terms were “inflammatory response,” “positive regulation of cell migration” and “signal transduction” (Figure S3C, Supporting Information). A heatmap of co‐differentially regulated factors in various pathways is presented in Figure 3C. “Cytokine−cytokine receptor interaction” was highly enriched. And previous studies suggested that cytokines and chemokines are critical factors in inducing immune cells recruitment and activation.^[^ ^15^ ^]^ This raises the possibility that aberrant expression of cytokines and chemokines may contribute to the role of PRP19 in B‐cell function and HCC development.

To identify the key factor in modulating B cell function, we assessed the migration properties of B cells treated with different cytokines using a Transwell assay. We found that CXCL12 increased B cell recruitment in the lower chamber, and other cytokines had little recruitment effect on B cells (Figure 3D). To validate the RNA sequencing results, we performed qPCR and ELISA assays and found that CXCL12 expression was upregulated in human and mouse PRP19 knockdown HCC cells (Figure 3E,F) and down‐regulated in PRP19 overexpressed HCC cells (Figure S3D, Supporting Information). Pearson analysis showed that CXCL12 expression was negatively correlated with PRP19 in TCGA‐LIHC (R = −0.28, p = 7.9e‐11) (Figure 3G) and Gao's Cohort (R = −0.29, p < 0.0001) (Figure S3E, Supporting Information). CXCL12 RNA levels were lower in HCC tissues than in normal liver and adjacent tumor tissues (Figure S3F,G, Supporting Information), and low expression of CXCL12 indicated poor clinical outcomes in patients with HCC (Figure S3H, Supporting Information).

CXCL12 has potential chemotactic activity for lymphocytes, and previous studies have reported that CXCL12 plays a critical role in modulating B cell development and activation in various diseases.^[^ ^16^ ^]^ Previous studies have indicated that CXCL12 exerts its biological function by specifically interacting with the ligand for the transmembrane G protein‐coupled receptor CXCR4 to activate multiple intracellular signaling pathways, such as ERK1/2 and MAPK.^[^ ^17^ ^]^ Recently, Xu et al. reported that CXCL12 can activate B cells to differentiate into immature plasma cells with IgG production to inhibit colorectal cancer liver metastasis.^[^ ^18^ ^]^

**Figure 4:**
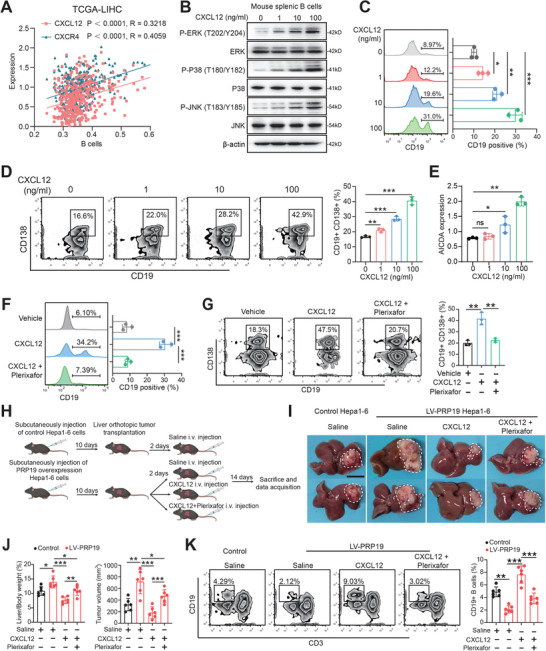
CXCL12 promotes B cell function and suppresses HCC development. A) CXCL12 and CXCR4 were positively correlated B cell infiltration in TCGA‐LIHC dataset. B) Western‐blot analysis of MAPK pathway in B cell after treated by CXCL12. C) B cell migration ability was analyzed by FCM after being treated by CXCL12. D) Plasma cell differentiation was explored by FCM and quantified when treated with CXCL12. E) AICDA expression levels in B cells were analyzed by qPCR when treated with CXCL12. (F) B cell migration ability was analyzed by FCM after being treated by CXCL12 with or without CXCR4 inhibitor, plerixafor. G) Plasma cell differentiation was explored by FCM and quantified after being treated by CXCL12 with or without plerixafor. H) Schematic for construction of mouse orthotopic liver tumor model. I) The liver images of mouse orthotopic tumors in different groups. J) The mouse liver, body weights of the mouse, and tumor volume in each group were measured at the endpoint. K) The infiltration levels of CD19+ B cell in mouse tumors were analyzed by FCM. *p < 0.05, **p < 0.01, ***p < 0.001 by student's t‐test (C, D, E), Pearson correlation analysis, (A) and ANOVA test (F, G, J, K). Abbreviation: LV‐PRP19, lentivirus‐PRP19.

First, we investigated the expression of CXCL12 and CXCR4 in HCC microenvironment using published single‐cell sequencing data, which included myeloid cells (CD14 and CD68), T cells (CD3D and CD3G), B cells (CD79A and MS4A1), malignant cells (AFP and GPC3), endothelial cells (PECAM1 and CDH5).^[^ ^19^ ^]^ B cells displayed the most abundant expression of CXCR4, and a high level of CXCR4 was also shown in myeloid cells and T cells. CXCL12 was mainly expressed by malignant cells and moderately expressed by endothelial cells (Figure S4A, Supporting Information). To clarify the relationship between the CXCL12/CXCR4 axis and B cells in HCC development, we calculated the B cell infiltration scores of HCC samples in the TCGA‐LIHC dataset using the ssGSEA algorithm. Notably, B‐cell infiltration scores were positively correlated with the expression of CXCL12 (R = 0.3218, p < 0.0001) and CXCR4 (R = 0.4059, p < 0.0001) (**Figure** **4**A). We collected mouse splenic B cells using specific magnetic beads (Figure S4B, Supporting Information) and treated them with different concentrations of CXCL12. CXCL12 promoted the phosphorylation of ERK (T202/Y204), P38 (T180/Y182), and JNK (T183/Y185) in mouse splenic B cells (Figure 4B). The migration assay showed that CXCL12 increased B cell recruitment in a dose‐dependent manner (Figure 4C). Exogenous CXCL12 promoted CD138+ plasma cell differentiation (Figure 4D). Activation‐induced cytidine deaminase (AICDA) is known as a key regulator in B cell activation and plasma cell development.^[^ ^20^ ^]^ Here, we found that AICDA expression was elevated in B cells after CXCL12 treatment (Figure 4E). We further explored B cell migration and plasma cell differentiation when treated with the CXCR4 inhibitor plerixafor and found that blocking CXCR4 by plerixafor could inhibit B cell recruitment and plasma cell differentiation ability mediated by CXCL12 in vitro (Figure 4F,G). Moreover, we co‐cultured mouse B cells with HCC cells and investigated B cell subtypes, including mature B cells (CD19+B220+CD22+), germinal center B cells (CD19+ B220+CD38+), memory B cells (CD19+ B220+CD27+ CD138‐), and plasma cells (CD19+ B220‐/low CD138+).^[^ ^21^ ^]^ We found that the proportion of plasma cells was significantly increased when co‐cultured with PRP19 knockout HCC cells; nevertheless, mature B, germinal center B, and memory B exhibited fewer differences (Figure S4C,D, Supporting Information). In the TCGA‐LIHC dataset, the PRP19‐high group had a lower plasma cell ratio than the RPR19‐low group, which supports our early findings (Figure S4E, Supporting Information).

To validate the role of CXCL12 in modulating B cell functions in vivo, we applied a mouse liver orthotopic xenograft model using stably overexpressing PRP19 HCC cells and treated them with the CXCL12 and CXCR4 inhibitor plerixafor (Figure 4H). The results showed that PRP19 overexpression promoted HCC development in a mouse orthotopic xenograft model and administration of CXCL12 suppressed HCC progression in the PRP19 overexpression group. Blocking CXCR4 impaired the anti‐tumor effect of CXCL12 in a mouse model (Figure 4I,J). Moreover, we investigated the infiltration levels of B cells in mouse liver xenografts and identified a lower proportion of B cells in the PRP19 overexpression group when compared with the control group. Administration of CXCL12 rescued the B cell infiltration level in the PRP19 overexpression mouse model, but this increase was impeded by the CXCR4 inhibitor (Figure 4K). Collectively, these findings suggest that PRP19 deficiency promotes CXCL12 expression and further activates B cell function via the receptor CXCR4 to suppress HCC pathogenesis.

### 2.4 CXCR4+ B Cells Inhibit HCC Pathogenesis with Enhanced Infiltration of NKs and Macrophage

**Figure 5:**
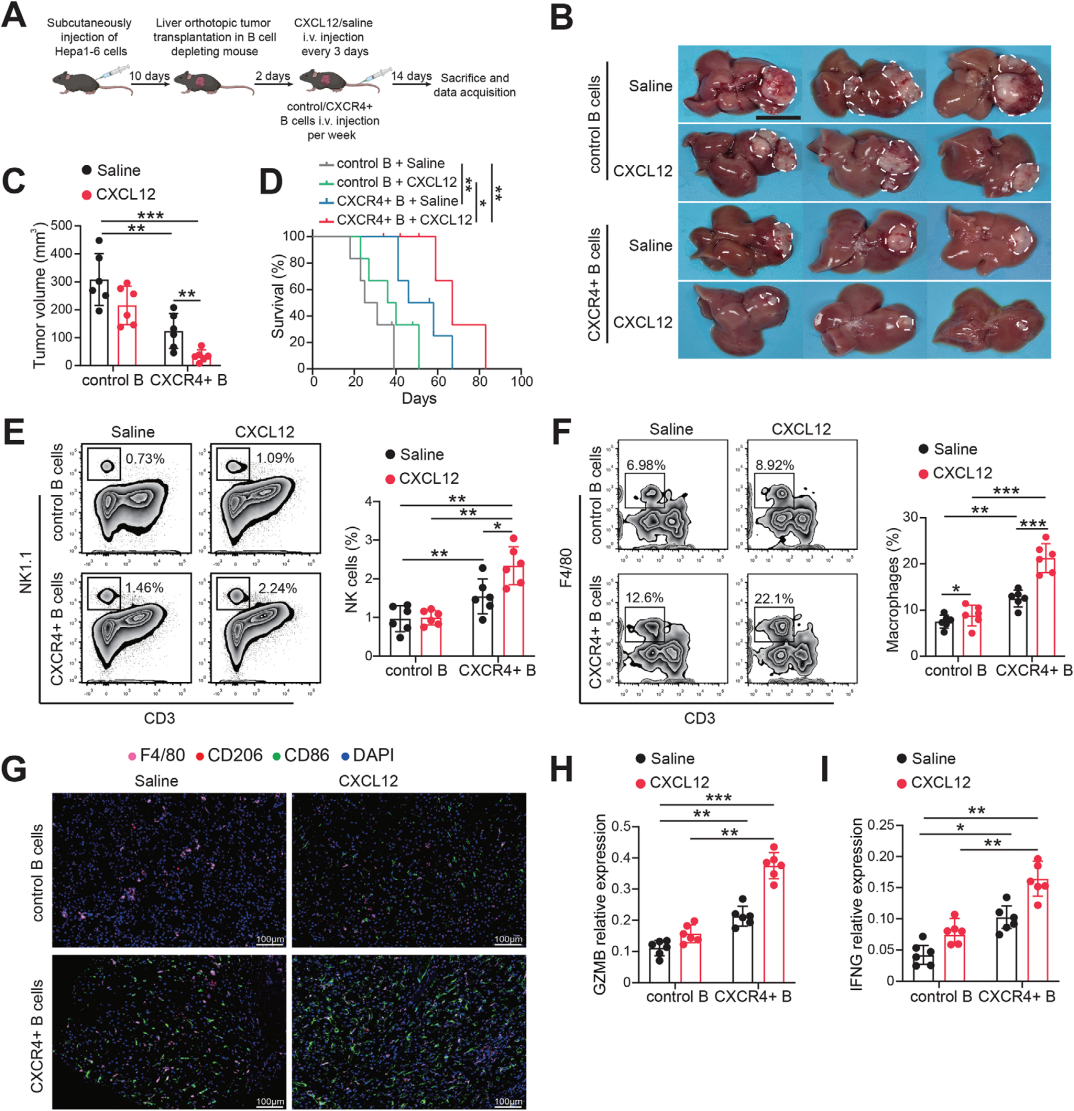
CXCR4+ B cells suppress mouse HCC development in vivo. A) Schematic for construction and treatment of mouse orthotopic liver tumor model. B) The liver images of mouse orthotopic tumors in different treatment groups. C) The tumor volume in each group was measured at the endpoint. D) The survival plot of each treatment group was analyzed by Kaplan–Meier method. The infiltration level of NK cells E) and macrophages F) in mouse tumors was explored by FCM. G) mIF staining of F4/80, CD206, CD86 in mouse HCC tissues. GZMB H) and IFNG I) expression in each group of mouse HCC tissues were analyzed by q‐PCR. *p < 0.05, **p < 0.01, ***p < 0.001 by ANOVA test (C, E, F, H, I) and log‐rank (Mantel‐Cox) test (D). mIF, multiple immunofluorescence.

The enhanced biological functions of B cells controlled by CXCL12 and its receptor CXCR4 prompted us to evaluate whether the adoptive transfer of CXCR4+ B cells is an optional and feasible immunotherapy for HCC. First, we isolated B cells from mouse spleens and overexpressed CXCR4 using lentivirus ex vivo (Figure S5A,B, Supporting Information). Furthermore, we constructed an orthotopic tumor in the mouse liver after depleting B cells with an anti‐CD20 antibody. These tumor‐bearing mice were adoptively transferred with control or CXCR4+ B cells and saline or CXCL12 (**Figure** **5**A). We found that the adoptive transfer of CXCR4+ B cells combined with CXCL12 effectively inhibited HCC progression (Figure 5B,C) and prolonged the survival time of tumor‐bearing mice (Figure 5D). Antibodies secreted by terminally differentiated plasma cells are critical for immune defense in the tumor microenvironment, which labels tumor cells and induces macrophage‐ and NK‐cell‐dependent malignant cell‐killing effects.^[^ ^5^ ^b,^ ^22^ ^]^ We observed increased infiltration levels of NK cells (Figure 5E), and macrophages (Figure 5F) in HCC tissues upon adoptive transfer of CXCR4+ B cells combined with CXCL12 treatment. To further the detail types of recruited macrophage, we performed the multiple immunofluorescence (mIF) in mouse HCC tissues with staining M1 marker (CD86) and M2 marker (CD206). Results showed that M1 macrophage infiltration was highly increased by administrating CXCR4+ B cells and CXCL12 in mice HCC model (Figure 5G). Besides, cytotoxic factor expressions, such as GZMB and IFNG, were up‐regulated in combination treatment mice, indicating prominent tumor cell‐killing ability (Figure 5H,I). Correlation analysis revealed that CXCL12 was positively correlated with NCAM1 (CD56), CD86, GZMB, and IFNG in the TCGA‐LIHC dataset (Figure S5C–F, Supporting Information). These results suggest that CXCR4+ B cells can be activated by CXCL12 to remodel the TME by recruiting NKs and M1 macrophages to inhibit HCC tumorigenesis.

### 2.5 DDX5 Regulates CXCL12 mRNA Expression by Stabilizing its Transcript

**Figure 6:**
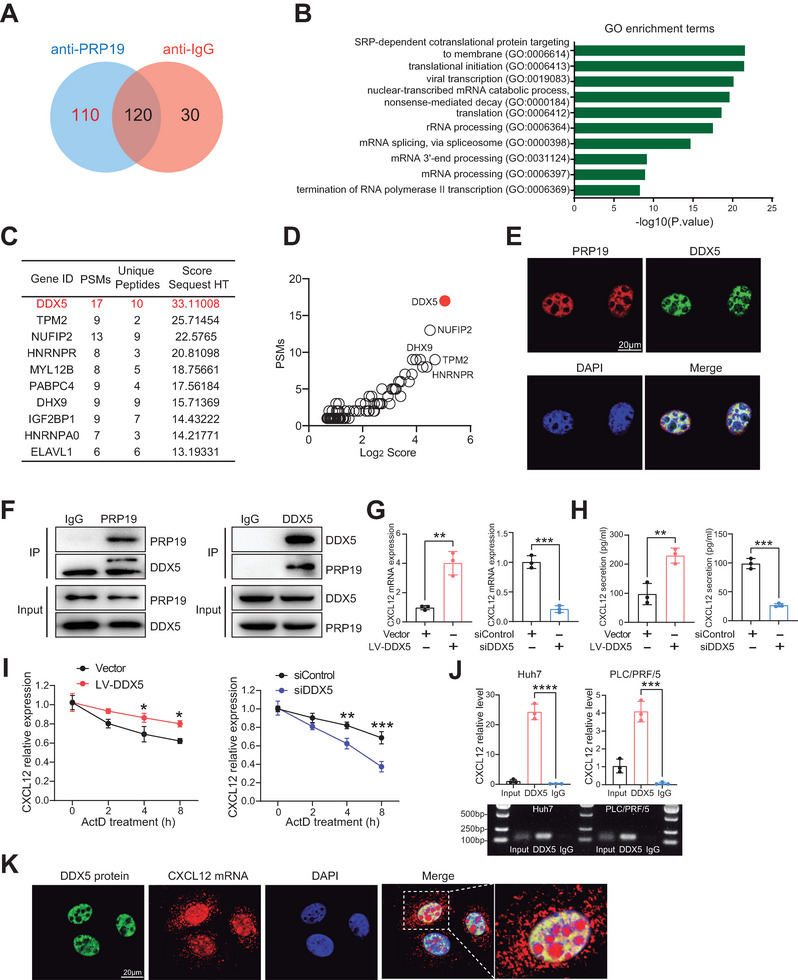
DDX5 regulates CXCL12 mRNA stability. A) Identifying PRP19 interacted proteins by LC‐MS/MS analysis. B) GO enrichment analysis of identified candidates. C) TOP10 identified candidates who interacted with PRP19. D) DDX5 was highly identified to interact with PRP19. E) Cellular co‐location of PRP19 and DDX5 was explored in HCC cells by IF staining. F) Co‐IP of endogenous PRP19 and DDX5 in HCC cells. G) CXCL12 mRNA level was analyzed by qPCR in DDX5 overexpression and knockdown HCC cells. H) CXCL12 protein level was analyzed by ELISA in DDX5 overexpression and knockdown HCC cells. I) The CXCL12 mRNA stability was explored in DDX5 overexpression and knockdown HCC cells. J) RIP assay was performed to explore the interaction of DDX5 protein and CXCL12 mRNA. K) Cellular co‐location of DDX5 protein and CXCL12 mRNA were explored in HCC cells by IF and FISH staining. *p < 0.05, **p < 0.01, ***p < 0.001 by student's t‐test (G, H, I, J).

To further clarify the potential regulatory mechanism of CXCL12 by PRP19, we performed LC‐MS/MS to identify target proteins interacting with PRP19. A total of 110 candidates were identified (**Figure** **6**A). GO enrichment analysis suggested that the 110 identified proteins were mainly associated with RNA catabolism and processing (Figure 6B). The top 10 interacting proteins are listed in Figure 6C, and DEAD‐box helicase 5 (DDX5) had the highest score (Figure 6D), suggesting that DDX5 may interact with PRP19 to intervene in subsequent biological processes. IF staining showed that PRP19 and DDX5 were colocalized in the nuclei of HCC cells (Figure 6E). Reciprocal co‐immunoprecipitation (Co‐IP) assays confirmed that PRP19 interacted with DDX5 (Figure 6F). To investigate whether DDX5 regulates CXCL12 expression, we exogenously altered DDX5 expression in HCC cells (Figure S6A,B, Supporting Information). We found that DDX5 knockdown suppressed CXCL12 expression, whereas DDX5 overexpression had the opposite effect (Figure 6G,H). In the TCGA‐LIHC dataset, CXCL12 positively correlated with DDX5 expression (R = 0.27, p = 2.6e‐09) (Figure S6C, Supporting Information). Nevertheless, CXCL12 expression was not altered when the levels of the other identified candidates were changed (Figure S6D–F, Supporting Information).

Previous studies have established that as an RNA‐binding protein, the molecular functions of DDX5 are mainly involved in transcriptional regulation, such as RNA alternative splicing.^[^ ^23^ ^]^ Several transcription isoforms of CXCL12 have been detected in biological and pathological processes.^[^ ^24^ ^]^ CXCL12‐α, CXCL12‐β, and CXCL12‐γ are the three predominant isoforms among the CXCL12 splice variants and have different biological functions. CXCL12‐α is the most widespread splicing variant and can manage the hematopoietic stem cell (HSC) population in the bone marrow.^[^ ^25^ ^]^ CXCL12‐β is associated with angiogenic properties.^[^ ^26^ ^]^ CXCL12‐γ is mainly localized within the nucleus and has potent anti‐HIV activity.^[^ ^27^ ^]^ To examine whether DDX5 regulates CXCL12 RNA alternative splicing, we assessed CXCL12‐α, CXCL12‐β, and CXCL12‐γ expression levels in DDX5 overexpression and knockdown HCC cells. We found that changes in DDX5 expression had no significant effect on CXCL12 RNA splicing (Figure S6G,H, Supporting Information). Moreover, several studies have reported that DDX5 regulates the transcriptional processing of target genes by maintaining RNA stability.^[^ ^23^ ^b,^ ^28^ ^]^ Therefore, to assess the impact of DDX5 on CXCL12 mRNA stability, we inhibited RNA transcription using Actinomycin D (ActD) and examined CXCL12 mRNA levels in DDX5 overexpression and knockdown HCC cells. We found that knockdown of DDX5 resulted in decreased stability of CXCL12 transcript, which showed an attenuated half‐life time compared with control cells, and overexpression of DDX5 displayed an inverse impact (Figure 6I). The RIP assay showed CXCL12 enrichment in DDX5 immunoprecipitation (IP) compared to the negative control, IgG (Figure 6J). Moreover, the interaction between DDX5 protein and CXCL12 mRNA was confirmed by IF and FISH co‐staining (Figure 6K). These results suggested that DDX5 acts as a critical mediator of PRP19 by directly interacting with CXCL12 mRNA to stabilize its mature transcript.

### 2.6 PRP19 Interacts with DDX5 to Induce the Degradation of DDX5 Protein

**Figure 7:**
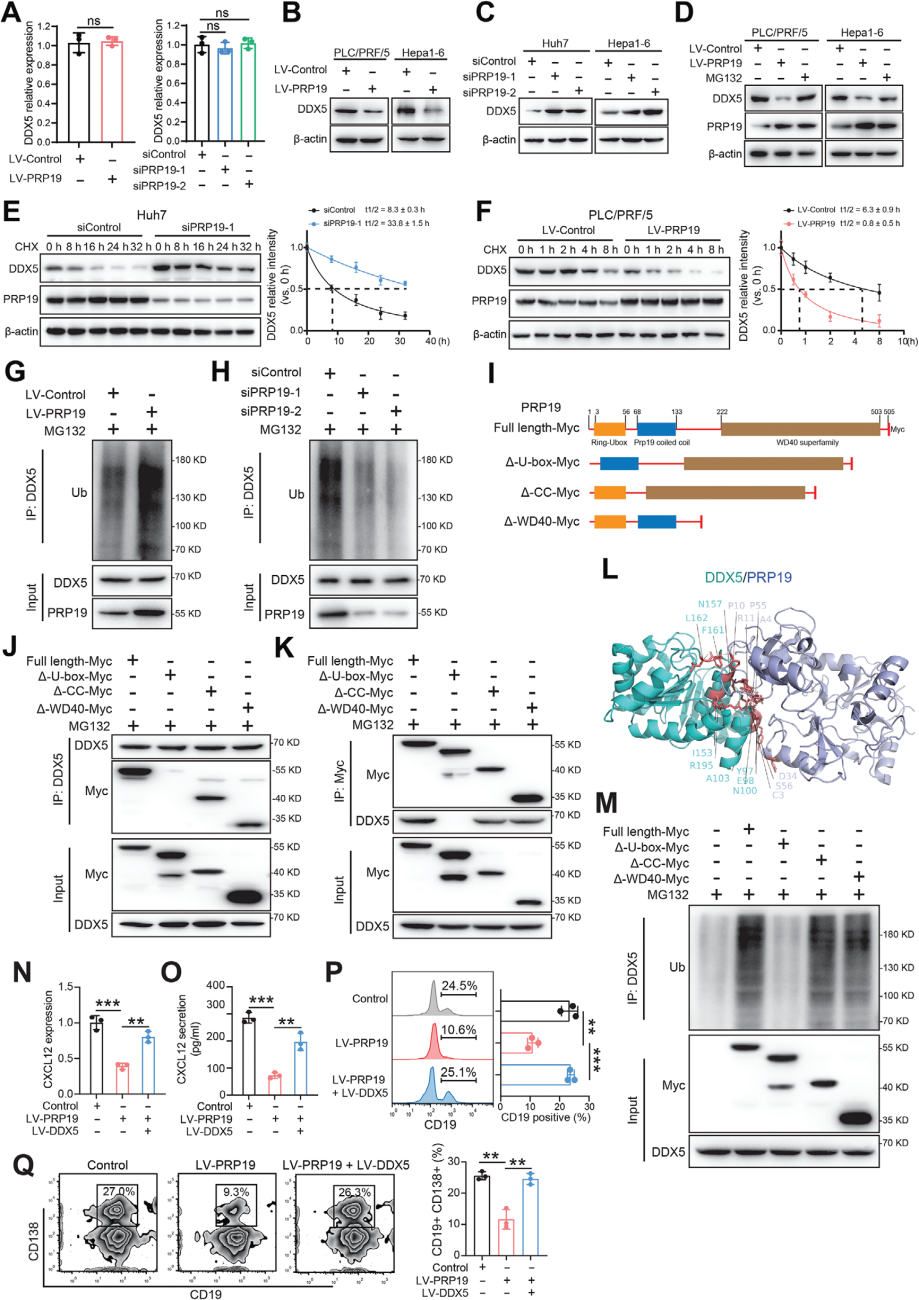
PRP19 mediates DDX5 protein degradation to regulate CXCL12 expression. A) DDX5 mRNA level was analyzed in PRP19 overexpression and knockdown HCC cells by qPCR. DDX5 protein level was analyzed in PRP19 overexpression B) and knockdown C) HCC cells by Co‐IP blot. D) DDX5 protein level was analyzed in PRP19 overexpression HCC cells when treated with MG132. E) DDX5 protein degradation half‐time was investigated in PRP19 overexpression human HCC cells. F) DDX5 protein degradation half‐time was investigated in PRP19 knockdown human HCC cells. DDX5 protein ubiquitination was analyzed in PRP19 overexpression G) and knockdown H) HCC cells by Co‐IP assay. I) PRP19 domain mutation plasmids were constructed. HEK293T cells were transfected with indicated PRP19‐Myc plasmids and cell lysates were Co‐IP with anti‐DDX5 J) and anti‐Myc K) Abs. L) The binding sites between PRP19 and DDX5 were predicted using ZDOCK tool. M) DDX5 protein ubiquitination was analyzed in HEK293T cells transfected with indicated PRP19‐Myc plasmids. CXCL12 mRNA N) and protein O) levels were analyzed in PRP19 overexpression HCC cells with or without DDX5 overexpression. P) B cell migration was explored when co‐cultured with PRP19 overexpression HCC cells with or without DDX5 overexpression. Q) Plasma cell differentiation was explored when co‐cultured with PRP19 overexpression HCC cells with or without DDX5 overexpression. Ns, not significant; **p < 0.01, ***p < 0.001 by student's t‐test (A) and ANOVA test (N, O, P, Q). Abbreviation: CHX, cycloheximide.

The LC‐MS/MS and Co‐IP results indicated that PRP19 could interact with DDX5 at the protein level. Therefore, we investigated whether PRP19 affects DDX5 expression. The mRNA level of DDX5 showed no significant changes upon PRP19 overexpression or knockdown of HCC cells of humans and mice (**Figure** **7**A; Figure S7A, Supporting Information). Nevertheless, we found that DDX5 protein levels were decreased when PRP19 was overexpressed (Figure 7B) but increased when PRP19 was knocked down in human and mouse HCC cells (Figure 7C). Previous studies have suggested that PRP19 could act as an E3 ubiquitin ligase that enables E3 activity in the ubiquitin‐proteasome system.^[^ ^29^ ^]^ Therefore, we hypothesized that PRP19 inhibits DDX5 protein levels via the ubiquitin‐proteasome system. By treating with the proteasome inhibitor, MG132 in PRP19 overexpressed HCC cells, we found that attenuated DDX5 protein levels were rescued (Figure 7D). Silencing PRP19 enhanced the half‐life of DDX5 degradation from 8.3 to 33.8 h in Huh7 cells (Figure 7E). PRP19 markedly reduced the half‐life of DDX5 degradation from 6.3 to 0.8 h in PLC/PRF/5 cells (Figure 7F). Similar protein degradation results for DDX5 were found in mice Hepa1‐6 cells (Figure S7B,C, Supporting Information). In addition, the Co‐IP assay showed that overexpression of PRP19 promoted DDX5 ubiquitination (Figure 7G), and silencing PRP19 inhibited DDX5 ubiquitination (Figure 7H). This suggests that PRP19 is a key regulator of DDX5 ubiquitination and degradation. PRP19 has three domains: an N‐terminal U‐box, a central coiled‐coil, and a C‐terminal WD‐40 repeat domain.^[^ ^30^ ^]^ To determine which domain of PRP19 interacts with DDX5, we constructed three domain mutation plasmids of PRP19 (Figure 7I). We transfected the mutant plasmids into HEK293T cells and performed a Co‐IP assay. The results showed that the binding between PRP19 and DDX5 was interrupted when the PRP19 N‐terminal U‐box domain mutation (Figure 7J,K). We then performed a computational 3D complex structural model using ZDOCK based on the X‐ray crystal structure of PRP19 and DDX5. Protein docking data showed that amino acids C3, A4, P10, R11, D34, P55, S56 of PRP19 U‐box domain, as well as amino acids Y97, E98, N100, A103, I153, N157, F161, L162, R195 of DDX5 were responsible for the interaction (Figure 7L). Moreover, the PRP19 U‐box domain mutation attenuated DDX5 ubiquitination (Figure 7M). These results indicate that the N‐terminal U‐box of PRP19 interacts with DDX5 to mediate its ubiquitination and degradation of DDX5.

We further examined whether PRP19 could manipulate CXCL12 expression and B cell function via DDX5. By upregulating DDX5 in PRP19 overexpression HCC cells, we found that CXCL12 expression increased (Figure 7N,O). Overexpression of DDX5 rescued the blunted migration ability of B cells and plasma cell differentiation when co‐cultured with PRP19 overexpression HCC cells (Figure 7P,Q). Moreover, silencing DDX5 in PRP19 knockdown HCC cells inhibited CXCL12 levels (FigureS7D,E, Supporting Information), B cell migration (Figure S7F, Supporting Information) and plasma cell differentiation (Figure S7G, Supporting Information). These data suggest that DDX5 is a key mediator of PRP19 to regulate CXCL12 expression and B cell function in HCC development.

### 2.7 Levels of PRP19, DDX5 and CD20+ B Cell were Correlated in HCC Tissues and Indicated Prognosis for HCC Patients

**Figure 8:**
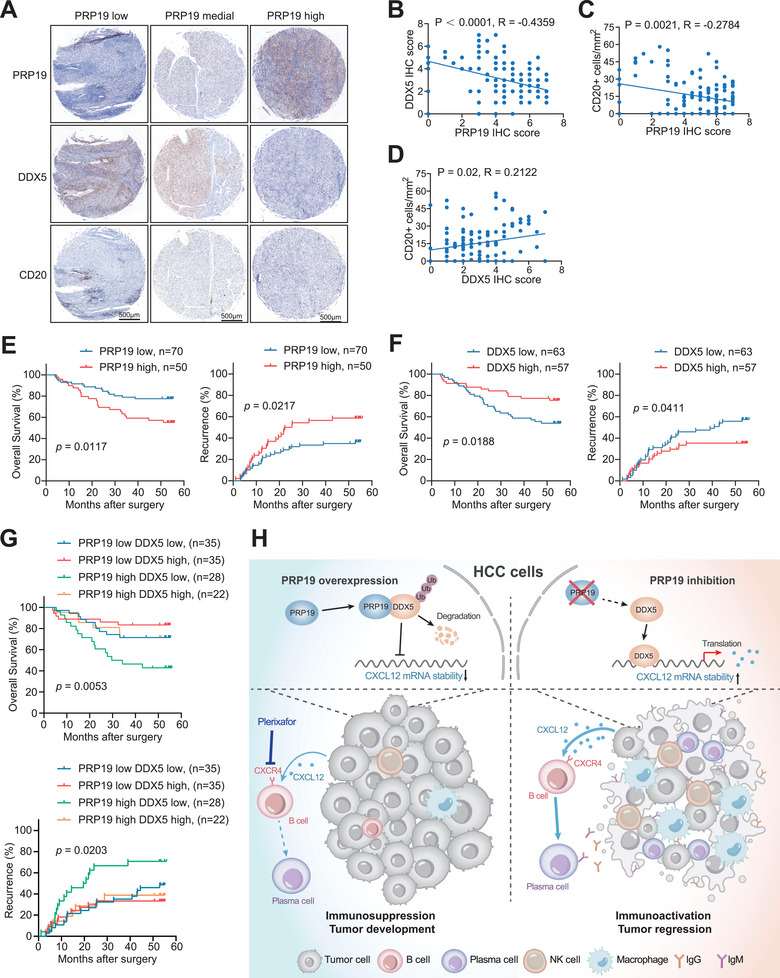
Levels of PRP19, DDX5 and CD20+ B cell in HCC tissues were indicated prognostic value. A) Representative IHC staining images for DDX5 and CD20 in PRP19‐low, ‐medial, and ‐high HCC samples obtained from Zhongshan Hospital, Fudan University. B–D) Correlation analysis of PRP19, DDX5, and CD20+ B cell levels in HCC samples. Kaplan‐Meier survival analysis showing overall survival and cumulative recurrence rates of PRP19 E) and DDX5 F). G) Kaplan‐Meier survival analyzed the prognostic value combining PRP19 and DDX5. H) Schematic model for the role of PRP19 in meditating B cell immune function during HCC development. Significance was determined by Pearson correlation analysis (B, C, D) and log‐rank (Mantel‐Cox) test (E, F, G).

We measured the expression of PRP19 and DDX5 and the infiltration of CD20+ B cells in a tissue microarray containing 120 HCC samples obtained from Zhongshan Hospital. The clinical features of HCC samples are presented in Table S2 (Supporting Information). Representative images of IHC staining are shown in **Figure** **8**A. Patients with HCC were divided into PRP19‐low, ‐medial, and ‐high groups based on the H‐score. Patients in the PRP19‐low group had higher DDX5 expression and CD20+ B‐cell infiltration. Nevertheless, HCC patients with high PRP19 expression had lower DDX5 expression and CD20+ B cell infiltration (Figure 8A). Correlation analysis suggested that PRP19 was negatively correlated with DDX5 (R = 0.2122, p = 0.02, Figure 8B) and CD20 (R = −0.4359, p < 0.0001, Figure 8C) expression in HCC patients, and DDX5 was positively associated with CD20 levels (R = −0.2784, p = 0.0021, Figure 8D). Kaplan‐Meier survival analysis showed that high expression of PRP19 indicated a poor overall survival rate and elevated tumor recurrence in HCC patients (Figure 8E). Conversely, patients with low DDX5 levels had poor clinical outcomes and high recurrence rates after surgery (Figure 8F), which is consistent with an earlier study.^[^ ^31^ ^]^ Moreover, we found that patients with high PRP19 and low DDX5 levels had the worst prognostic outcomes among all four groups (Figure 8G). Collectively, our data revealed that PRP19 plays a critical role in B cell immune dysfunction via mediating DDX5 and CXCL12 levels, and is clinically associated with poor prognosis of HCC (Figure 8H).

## 3 Discussion

The tumor microenvironment (TME) is composed of an extracellular matrix and various types of cells, including malignant, stromal, and immune cells.^[^ ^32^ ^]^ Recently, the success of immune checkpoint inhibitors has provided a potent tool for human malignancy treatment, and the function of the immune microenvironment in HCC progression has been extensively investigated.^[^ ^15^ ^a,^ ^32^ ^a]^ However, significant attention has been focused on T cells and myeloid cells in the immune response, and the immune feature and recruitment mechanism of tumor‐infiltrating B cells remains relatively understudied.^[^ ^5^ ^b,^ ^33^ ^]^ In this study, we divided HCC into B cell high and low infiltration groups according to the B cell scores. By analyzing the expression profiling between two groups, we identified that PRP19 expression was decreased in B cell high infiltrated HCC samples, and negatively correlated with B cell marker (CD20).

In early studies, we have reported the key functions PRP19 in tumorigenesis by modulating the cell cycle, repairing DNA damage, and increasing traditional therapeutic resistance.^[^ ^11^, ^34^ ^]^ Nevertheless, the biological pattern of PRP19 in the TME of HCC has yet to be elucidated. Using flow cytometry (FCM) analysis, we identified that PRP19 was mainly expressed in non‐immune cells in HCC, and CD19+ B cells were highly infiltrated in the PRP19‐low group patients. In vivo mouse model and in vitro 3D spheroids assays suggested that B cell recruitment ability was increased in PRP19 inhibition HCC cells. These findings indicate PRP19 novel role for B cell regulation in HCC development.

The CXCL12/CXCR4 axis acted as pleiotropic pro‐oncogenic and anti‐oncogenic effects rely on the cellular context and microenvironment in tumorigenesis. Early researchers reported that CXCR4 expression in lung cancer could serve as a marker of cancer‐initiating cells and be associated with poor clinical outcomes.^[^ ^35^ ^]^ In pancreatic ductal adenocarcinoma, high levels of CXCL12 expressed by carcinoma‐associated fibroblasts (CAFs) lead to immunosuppression and T‐cell dysfunction.^[^ ^36^ ^]^ However, other investigators have shown the antitumor effect of the CXCL12/CXCR4 axis. The interaction between CXCL12 and CXCR4 induces CD47 internalization and promotes tumor cell phagocytosis by macrophages in mesothelioma tumors.^[^ ^37^ ^]^ Exogenous treatment of cervical cancer cells with CXCL12 in vitro inhibited cell metastasis and growth events.^[^ ^38^ ^]^ Besides, High CXCL12 expression was associated with better prognostic outcomes in breast cancer and endometrial cancer patients.^[^ ^39^ ^]^ In the present study, we found that the CXCL12/CXCR4 axis is critical for PRP19 meditated B cell enrichment in HCC. Knockdown of PRP19 in HCC cells promoted CXCL12 expression, which interacted with CXCR4 to cause B‐cell recruitment and plasma cell development, supporting the establishment of a potent antitumor immune response. Administering CXCL12 in vivo inhibited mouse HCC progression, and this anti‐tumor effect could be abrogated by a CXCR4 inhibitor. Remarkably, early studies found that CXCR4 is expressed on the surface of B cells and mediates their recruitment and development,^[^ ^18^, ^40^ ^]^ which supported our findings.

Several studies suggested that plasma cells with specific immunoglobulin isotypes can have differing pro‐tumor and anti‐tumor roles in tumor microenvironments.^[^ ^6^, ^22^, ^41^ ^]^ Given that antibody‐dependent cell‐mediated cytotoxicity (ADCC) is a direct mechanism of the anti‐tumor response mediated by plasma cells, therapeutic approaches that recover functional antibody isotypes to support this mechanism should be generally beneficial for HCC patients. Here, we identified that the adoptive transfer of CXCR4+ B cells combined with exogenous CXCL12 treatment could elevate NK cells and macrophage infiltration to suppress mouse HCC development via ADCC response, which provides a promising immunotherapy option for clinical HCC treatment. Systemic administration is widely used in both basic research and clinical cancer immunotherapy due to its convenience. However, since elevated CXCL12 levels could enhance B cell migration, systemic administration of exogenous CXCL12 might partially counteract the chemotactic effect of tumor‐secreted CXCL12. In contrast, localized intratumoral administration offers distinct advantages in improving therapeutic efficacy and reducing systemic side effects, making it a viable option for treating HCC.

DDX5 is a member of the DEAD‐box RNA helicase paralogs that mainly mediates RNA processing, transcription, and splicing.^[^ ^23^ ^a]^ In HCC, researchers identified that DDX5 is downregulated and associated with poor clinical outcomes.^[^ ^23^ ^c,^ ^31^, ^42^ ^]^ Sun et al. reported that DDX5 resolves the G‐quadruplex structure of STAT1 mRNA to promote STAT1 translation and maintain the antiviral effect of IFN‐α in HBV‐related HCC.^[^ ^42^ ^a]^ In addition, DDX5 induces autophagy and suppresses HCC tumorigenesis by interacting with the autophagic receptor p62, which is independent of its RNA‐binding and helicase activity.^[^ ^31^ ^]^ Here, we found that the U‐box domain of PRP19 interacts with DDX5 and promotes DDX5 protein degradation by the ubiquitin‐proteasome system in HCC. DDX5 is a key regulator for maintaining CXCL12 mRNA stability to increase CXCL12 expression but does not affect its RNA splicing event. As early studies reported, DDX5 protein regulates CXCL12 expression through several potential mechanisms, such as facilitating CXCL12 pre‐mRNA maturation, influencing RNA modification, and protecting CXCL12 mRNA from RNA‐induced silencing complexes (RISC)‐mediated degradation.^[^ ^43^ ^]^ The mechanism underlying DDX5 stabilizing CXCL12 mRNA needs more in‐depth investigation. Moreover, overexpression of DDX5 could rescue the attenuated of CXCL12 expression and B cell function by PRP19. In a clinically independent HCC cohort, we found that PRP19 was negatively correlated with DDX5 and B cell infiltration, and high PRP19 expression and attenuated DDX5 expression were associated with poor prognosis in patients with HCC.

Due to tumor immune tolerance and the heterogeneity of the tumor microenvironment, several preclinical risks need to be addressed for the application of CXCL12 treatment and CXCR4+ B cell adoptive transfer. First, while our studies were conducted in mouse models, broader clinical trials are necessary to confirm the efficacy of these treatments in diverse patient populations. Second, establishing the optimal dosage and delivery method for CXCL12, as well as the appropriate number of cells for transfer, is crucial for ensuring patient safety and therapeutic success. Third, the long‐term effects of modulating the CXCL12/CXCR4 axis, including through adoptive transfer of CXCR4+ B cells and exogenous CXCL12 treatment, are not yet fully understood and require further investigation. Additionally, systemic administration of CXCL12 may lead to off‐target effects, such as counteracting tumor‐secreted CXCL12, highlighting the need for optimized delivery methods, such as localized administration, to improve therapeutic specificity and minimize side effects.

In conclusion, our study revealed a relationship between PRP19 expression and B cell function in HCC development. PRP19 inhibition promotes B cell recruitment to suppress HCC tumorigenesis via DDX5 mediated CXCL12‐CXCR4 axis. Moreover, we elucidated that combining the adoptive transfer of CXCR4+ B cells and CXCL12 treatment could effectively suppress HCC development in a mouse model by reshaping the TME. Thus, our work provides a rationale for immunotherapy for HCC by targeting PRP19 and modulating B‐cell immune function.

## 4 Experimental Section

### Clinical Samples

A total of 22 human HCC samples were obtained from Zhongshan Hospital, Fudan University. Fresh resected specimens were collected after surgery and stored in a tissue storage solution (Miltenyi Biotec, Koln, Germany) for flow cytometry (FCM) analysis (Table S1, Supporting Information). One HCC tissue microarray (TMA) was used in this study, which included 120 formalin‐fixed paraffin‐embedded HCC tissues and adjacent normal tissues (Table S2, Supporting Information). All patients with HCC underwent curative resection at the Liver Cancer Institute, Zhongshan Hospital, and written informed consent was obtained. This study was approved by the Research Ethics Committee of Zhongshan Hospital, Fudan University (B‐2020‐354R).

### Cell Lines

The human HCC cell lines Huh7 and PLC/PRF/5, mouse HCC cell line Hepa1‐6, and Human embryonic kidney cell line HEK‐293T were purchased from the Chinese Academy of Sciences Shanghai Branch Cell Bank (Shanghai, China). All the cell lines were cultured in Dulbecco's modified Eagle's medium (DMEM) (KeyGEN, Nanjing, China) supplemented with 10% fetal bovine serum (Sigma, Saint Louis, USA), 100 U mL^−1^ penicillin, and 100 mg mL^−1^ streptomycin (Gibco, California, USA), and maintained in a 37 °C humidified incubator with 5% CO2. All cell lines in this study were authenticated by short tandem repeat analysis and confirmed to be without mycoplasma contamination.

### Public Transcriptomic Datasets Analysis

The B cell infiltration differences were explored by ssGSEA R package in the HCC tissues expression profiling cohort (GSE101728), including 7 tumor samples and 7 para‐tumor tissues.^[^ ^13^ ^]^ The RNA sequencing data and clinical information of liver hepatocellular carcinoma (LIHC, *n* = 371) from TCGA (https://portal.gdc.cancer.gov/), Zhongshan Hospital HCC research (Gao's Cohort, OEP000321, *n* = 159)^[^ ^14^ ^]^ from the National Omics Data Encyclopedia (NODE, https://www.biosino.org/node), and HCC tissue sequencing data (GSE22058, *n* = 96) from GEO (https://www.ncbi.nlm.nih.gov/geo) were obtained to explore the expression and prognostic values of PRP19, CD20, CXCL12, and DDX5. Single‐cell sequencing data of HCC tissues was downloaded from CNP0000650 dataset^[^ ^19^ ^]^ in CNGBdb (China National Gene Bank Data Base) (https://db.cngb.org/) for immune microenvironment analysis.

### Flow Cytometry (FCM) Analysis

Tumor samples of humans or mice were cut into small pieces in 15 mL tubes and digested with collagenase IV (1 mg mL^−1^, Sigma, USA) and DNase I (Invitrogen, USA) for 1 h at 37 °C. The lytic tissue medium was then filtered using a 70 µm filter to obtain single‐cell suspensions. The cell suspensions were washed twice with PBS, stained with the indicated antibodies for 30 min on ice, and subjected to FCM analysis.

The following reagents and antibodies were used in FCM: LIVE/DEAD Fixable Stain (Invitrogen, California, USA), anti‐human PRP19‐PE (Santa Cruz, Texas, USA), anti‐human CD45‐BV510 (Biolegend, California, USA), anti‐human CD3‐BV396 (Biolegend, California, USA), anti‐mouse CD4‐FITC (BD, New Jersey, USA), anti‐mouse CD8‐PerCP‐Cy5.5 (Biolegend, California, USA), anti‐mouse CD115‐APC (Biolegend, California, USA), anti‐mouse CD45‐BV605 (Biolegend, California, USA), anti‐mouse CD19‐BV650 (Biolegend, California, USA), anti‐mouse CD20‐APC/cy7 (Biolegend, California, USA), anti‐mouse CD22‐APC (Biolegend, California, USA), anti‐mouse CD38‐PE/cy7 (Biolegend, California, USA), anti‐mouse CD138‐PE/Dazzle 594 (Biolegend, California, USA), anti‐mouse CD3‐PE/cy7 (Biolegend, California, USA), anti‐mouse F4/80‐PE (Biolegend, California, USA), anti‐mouse NK1.1‐PE (Biolegend, California, USA).

### Plasmids Construction and Cell Transfection

The CDS sequences of PRP19 and DDX5 were cloned into the pCDH‐CMV‐Myc and pCDH‐CMV‐HA lentiviral plasmids, respectively, for overexpression. The domain mutation of the PRP19 CDS was cloned into pEn‐CMV‐Myc for the Co‐IP assay. Knockout of human and mouse PRP19 was achieved by cloning sgRNA into the lentiCRISPR v2. Mouse CXCR4 lentiviral plasmid PMLV‐CMV‐CXCR4 and lentivirus particles were purchased from Genomeditech (Shanghai, China). Lentiviral plasmids were packaged using psPAX2 and pMD2G plasmids in HEK‐293T cells. The lentivirus particles were collected to infect subconfluent cultures with 5 µg/ml polybrene overnight. After 48 h, the cells were cultured in media with 2 µg/ml puromycin (Gibco, Carlsbad, CA, USA) to construct stable cell lines. PRP19, DDX5, DHX9, ELAVL1, and IGF2BP1 siRNAs were purchased from Tsingke Biotechnology (Shanghai, China). The siRNAs and recombinant plasmids were transfected into HCC cells using Lipofectamine3000 (Invitrogen, Carlsbad, CA, USA), according to the manufacturer's instructions. The sgRNA and siRNA sequences are listed in Table S3 (Supporting Information).

### Reverse Transcription and Quantitative PCR

Total RNA was extracted from cells using Trizol reagent (Takara, Japan), and the concentration of RNA was evaluated using a NanoDrop ONE UV spectrophotometer (ThermoFisher, California, USA). cDNA synthesis was performed with 1 µg of total RNA using a Hifair II 1st Strand cDNA Synthesis Kit (Yeasen, Shanghai, China). qRT‐PCR was performed using Hieff qPCR SYBR Green Master Mix (Yeasen, Shanghai, China) according to the manufacturer's instructions. The expression of β‐actin was selected as an internal control, and the relative expression levels of the target genes were determined using the 2^−ΔΔCT^ method. The PCR primers used in this study are listed in Table S4 (Supporting Information).

### Western‐Blot Assay

The indicated cells were lysed using RIPA buffer (NCM, Suzhou, China) containing a protease inhibitor cocktail (NCM, Suzhou, China) at 4 °C for 30 min, and centrifugated at high speed to obtain protein supernatants. Protein concentration was determined using a bicinchoninic acid (BCA) assay (WELLBI, Shanghai, China) according to the manufacturer's protocol. Proteins were separated by 12% SDS‐PAGE and transferred onto PVDF membranes (Millipore, Bedford, USA). The bands were incubated with the interested primary antibodies overnight at 4 °C, followed by the corresponding secondary antibody incubation at room temperature for 1 h. Protein bands were visualized using a chemiluminescence (ECL) imaging system (Tanon, Shanghai, China). The protein level of β‐actin was used as an internal control.

### Isolation of Peripheral Blood Mononuclear Cells and Mouse B Cells

Peripheral blood mononuclear cells (PBMC) were isolated from healthy donors using Ficoll density gradient centrifugation and cultured in RPMI 1640 medium supplemented with 10% fetal bovine serum. Mouse B cells were negatively selected from the spleen and lymphatic glands using the MojoSort Mouse Pan B Cell Isolation Kit (BioLegend, California, USA) following the manufacturer's instructions. The purity of the isolated B cells was evaluated using an anti‐mouse CD19‐BV650 (Biolegend, California, USA) using FCM analysis, which was routine>90%. Mouse B cells were cultured in RPMI 1640 medium supplemented with 10% fetal bovine serum and stimulated with IgG+IgM (5.4 µg mL^−1^, Jackson ImmunoResearch, USA), lipopolysaccharide (LPS, 10 µg mL^−1^, Beyotime, China), and mouse CD40L (1 µg mL^−1^, Absin, China).

### In Vitro B Cell Migration and Plasma Cell Differentiation Assays

Migration assays were performed using a Transwell system. PBMCs (1 × 10^6^) were added to the upper chamber, and PRP19 knockout or overexpression hepa1‐6 cells (5 × 10^4^) or indicated cytokines were added to the lower chamber. After 24 h, the cells in the lower chamber were harvested and CD19+ B cell ratios were determined by FCM analysis. For the plasma cell differentiation assay, mouse B cells were stimulated with IgG+IgM (5.4 µg mL^−1^, Jackson Immuno Research, USA), lipopolysaccharide (LPS, 10 µg mL^−1^, Beyotime, China), mouse CD40L (1 µg mL^−1^, Absin, China), and co‐cultured with the indicated hepa1‐6 cells or cytokines. After 48 h, the percentage of CD19+CD138+ plasma cells was analyzed using FCM.

### Enzyme‐Linked Immunosorbent Assay (ELISA)

Concentrations of CXCL12 in cell supernatants and mouse serum were analyzed using ELISA kits (eBioscience, USA) according to the manufacturer's instructions.

### 3D Spheroid Construction

3D HCC spheroids were constructed with control and PRP19 knockout hepa1‐6 cells (3000 cells per well) in 96‐well microplates (Corning, New York, USA) using 3D NACs‐origami assembly technique (Puheng Biotechnology, Su Zhou, China). Cell spheroids were cultured in DMEM medium with 10% FBS for 24 h. After that, the isolated mouse B cells (1 × 10^5^ cells per well) were seeded into the 3D spheroids and co‐cultured for 24 h. Immunofluorescence (IF) was applied using anti‐CD20 (1:100; Servicebio) to measure B cell infiltration, and the fluorescence intensity was quantified by ImageJ 1.5.4.

### Immunofluorescence (IF)

The indicated cells were seeded on coverslips in 24‐well plates. After 24 h, the cells were washed 3 times with PBS, fixed with 4% paraformaldehyde, permeabilized with Triton X‐100, and blocked with 5% bovine serum albumin for 1 h at room temperature. Then, the cells were incubated with primary antibodies: anti‐PRP19 (1:100; Santa Cruz), and anti‐DDX5 (1:100; Abclonal) overnight at 4 °C. The cells were then incubated with the corresponding secondary antibodies conjugated with Alexa Fluor −488 or −594 (1:200; Abclonal). The nuclei of the cells were stained with DAPI (WELLBI, Shanghai, China). Finally, stained cells were visualized using a laser confocal microscope (Leica TCS SP5 II, Wetzlar, Germany). For tissue IF, paraffin‐embedded slides were deparaffinized with xylene and rehydrated in ethanol. Then, the antigen was retrieved with citrate buffer and blocked with 5% goat serum for 30 min at 37 °C. The tissue slides were incubated with primary antibodies: anti‐PRP19 (1:100; Santa Cruz), anti‐CD20 (1:100; Servicebio), followed by incubation with the corresponding secondary antibodies. Finally, the slides were incubated with DAPI for nuclear staining. Images were captured using the CaseViewer software v. 2.4.

### Immunohistochemistry (IHC)

For the immunohistochemistry (IHC) assay, paraffin‐embedded human and mouse HCC tissues were cut into tissue sections, deparaffinized with xylene, and rehydrated in ethanol. The primary antibodies used for IHC staining were anti‐PRP19 (1:100, Santa Cruz,), anti‐DDX5 (1:100; Abclonal), anti‐CD20 (1:100; CST), anti‐CD56 (1:100, Abclonal), and anti‐F4/80 (1:100; Abclonal). For TMA staining analysis, we adopted the histological score (H‐score) approach to assess IHC staining as in a previous study.^[^ ^44^ ^]^ The H‐score of each sample was evaluated by two independent researchers who were blinded to the clinical features of the patients. The correlation between PRP19 expression and the clinical information of patients are listed in Table S3 (Supporting Information).

### RNA Sequencing

Total RNA from control and PRP19 knockdown HCC cells was extracted using Trizol reagent (Takara, Japan), and RNA purity and quality were analyzed using a 2100 Bioanalyzer (Agilent Technologies, California, USA). mRNA was enriched from total RNA samples by polyA purification, and cDNA was synthesized by reverse transcription using random primers. Library construction and sequencing were performed using PANOMIX Technology (Suzhou, China) using the Illumina sequencing platform. Across‐sample normalization and differential expression analyses were performed using the DESeq R package. Up‐ and down‐regulated genes were identified with |log2FoldChange| > 1 and *p* values <0.05. The GO and KEGG Pathways of the differentially expressed genes were enriched. RNA‐seq data in this study is deposited under the accession number CNP0004267 in the China National Gene Bank Data Base (CNGBdb, https://db.cngb.org/).

### Co‐Immunoprecipitation (Co‐IP) Assay

Cells were transfected with the indicated plasmids for 48 h, and cell pellets were lysed with immunoprecipitation (IP) lysis buffer (WELLBI, Shanghai, China). Then, the cell lysates were incubated with control IgG, and immunoprecipitating primary antibodies overnight at 4 °C with shaking. Protein A magnetic beads (40 µL; MedChemExpress, New Jersey, USA) were added to the cells and incubated for 2 h at room temperature. The magnetic beads were washed five times with IP lysis buffer and boiled at 100 °C for 10 min. Western‐blot was performed to detect interactions between the proteins.

### LC‐MS/MS

HCC cell lysates were IP with anti‐PRP19 (Santa Cruz, 1:100) and control anti‐IgG (Cell Signaling Technology, 1:100) overnight at 4 °C and incubated with Protein A magnetic beads (MedChemExpress, New Jersey, USA) for 2 h at room temperature. The protein interaction complexes were subjected to western‐blot and the protein bands in the gel were visualized using a silver staining kit (Beyotime, Shanghai, China). Protein bands were extracted, and mass spectrometric analysis was performed by OEbiotech (Shanghai, China).

### Protein Docking 3D Model Construction

The 3D structure PDB format files of PRP19 (ID: 2BAY) and DDX5 (ID: 3FE2) were downloaded from RCSB Protein Data Bank (https://www.rcsb.org/). The protein docking and binding sites prediction were analyzed using ZDOCK server.^[^ ^45^ ^]^ The PyMOL Molecular Graphics System v2.5 was applied to visualize the protein complex 3D model.

### RNA Stability Assay

The indicated HCC cells were cultured in 6‐well plates, and 5 µg mL^−1^ actinomycin D (ActD) was added at 0, 2, 4, and 8 h before cell scraping collection. Total RNA was isolated using TRIzol reagent (Takara, Japan), and qRT‐PCR was performed to quantify the relative levels of CXCL12 RNA between different groups.

### RNA Fluorescence In Situ Hybridization (FISH)

Cy3‐labeled CXCL12 mRNA‐specific FISH probes were designed and synthesized by RiboBio (Guangzhou, China). HCC cells were cultured on coverslips in 24‐well plates, fixed with 4% paraformaldehyde for 30 min, and permeabilized with 0.5% Triton X‐100 for 15 min. The FISH assay was performed using a FISH kit (BersinBio, Guangzhou, China) according to the manufacturer's instructions. The nuclei of the cells were stained with DAPI (; WELLBI, Shanghai, China). Images were obtained using a laser confocal microscope (Leica TCS SP5 II; Wetzlar, Germany).

### RNA Immunoprecipitation (RIP) Assay

The RIP assay was performed using an RIP kit (BersinBio, Guangzhou, China), according to the manufacturer's instructions. Briefly, 1 × 10^7^ HCC cells were collected and lysed using a polysome lysis buffer containing protease and RNase inhibitors. Then, the cell lysates were incubated with DDX5 or control IgG antibodies overnight at 4 °C, followed by protein A/G beads incubation for 2 h. Subsequently, immunoprecipitated RNAs were isolated and examined using qRT‐PCR and nucleic acid electrophoresis.

### Animal Study

C57BL/6 mice were purchased from Jackson Laboratory and maintained in a specific pathogen‐free (SPF) animal laboratory. The animal study was approved by the Animal Care and Use Committee of Zhongshan Hospital of Fudan University (2019‐073).

For mouse subcutaneous tumor model construction, indicated 1 × 10^7^ Hepa1‐6 cells were collected and resuspended in 150 µL PBS for each mouse. The cells were then subcutaneously injected into the right flank of the mice. Tumor growth was monitored every four days, and the mice were euthanized after 20 days. Tumor volumes were recorded and calculated using the formula: length (mm) × width^2^(mm) × 0.5. Tumor tissues were collected and labeled for subsequent analyses.

For mouse liver orthotopic tumor model construction, the indicated Hepa1‐6 cells were subcutaneously injected into the right flank of the mice. After 2 weeks, the subcutaneous tumors were cut, resected into 3 mm^3^ tissue pieces, and replanted into mouse livers to establish an orthotopic xenograft model. After 2 weeks, the mice were euthanized and their body and liver weights were recorded. Tumor tissues were collected and labeled for subsequent analyses. For mouse B cell deletion, an anti‐mouse CD20 antibody (10 µg g^−1^, Biolegend, California, USA) in 100 µL saline was intravenously injected into mice. B cell deletion efficiency was determined in the mouse spleen using FCM.

For the adoptive transfer experiments, purified mouse B cells were infected with mouse control or CXCR4 lentivirus particles to construct control or CXCR4+ B cells. Control or CXCR4+ B cells (1 × 10^7^) were adoptively transferred into B cells deleting orthotopic liver tumor mice per week. CXCL12 (BioLegend, California, USA) and saline buffer were administered every 3 days. After 2 weeks, the mice were euthanized, and data were collected.

### Statistical Analysis

All statistical analyses were performed using the SPSS software v21 (IBM, New York, USA) or GraphPad Prism v9 (California, USA). Bar graphs are presented as mean ± standard deviation (SD). Differences between multiple groups were compared using one‐way ANOVA, and multiple comparisons between two given groups were performed using the least significant difference (LSD) test. The student's t‐test was performed to analyze the differences between the two groups with a normal distribution. For abnormal distribution of variance between the two groups, the Mann‐Whitney U test was used to determine the difference. Pearson's correlation was adopted for the correlation analysis between 2 indicators. The survival curves were analyzed using the log‐rank (Mantel‐Cox) test for two or more groups and presented using the Kaplan–Meier method. All experiments were repeated at least thrice. Statistical significance was set at p < 0.05. ns, not significant; *p < 0.05, **p < 0.01, ***p < 0.001.

## Conflict of Interest

The authors declare no conflict of interest.

## Author Contributions

Z.L., X.L., D.Z., and D.G. contributed equally to this work. Z.L., X.S., R.X., and L.D. provided the concept and designed the study. X.L., D.Z., and D.G. collected the clinical HCC tissues. Z.L., X.L., D.Z., W.T., X.Y., and F.Z. performed experiments and analyzed the data. Z.L. and X.L. wrote the manuscript with the help of S.Z. D.G. provided TMA of clinical HCC tissues. S.Z., X.S., R.X., and L.D. contributed to manuscript preparation and finalization. All authors commented on and approved the manuscript.

## Supporting information



Supporting Information

## Data Availability

The data that support the findings of this study are available from the corresponding author upon reasonable request.
