# Supplementary material for: Increased PRP19 in Hepatocyte Impedes B Cell Function to Promote Hepatocarcinogenesis
Source: Adv Sci (Weinh). 2024 Oct 18;11(46):2407517. doi: 10.1002/advs.202407517 (PMC11633487; doi:10.1002/advs.202407517)
Supplement: Supplementary file 1 — Supporting Information [file ADVS-11-2407517-s001.docx]

# Supplementary figure legends


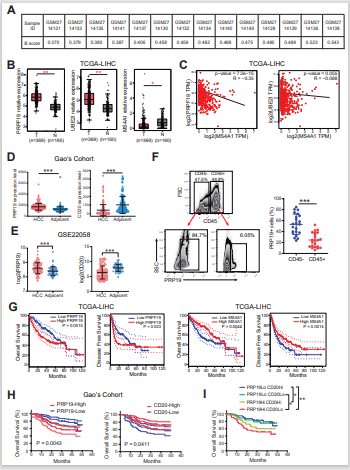


**Figure S1. Comparing the immune microenvironment differences between PRP19-low and -high HCC tissues.** (A) The B cell infiltration scores of each HCC sample in GSE101728. (B) TCGA-LIHC dataset showed that PRP19 and UBE2I expression was increased in HCC tissues, and MS4A1 was decreased in HCC tissues when compared with normal liver. (C) Correlation analysis of PRP19, UBE2I and MS4A1 in TCGA-LIHC dataset. (D) Comparing the expression of PRP19 and CD20 between HCC and adjacent tumor tissues from Gao’s cohort. (E) Comparing the expression of PRP19 and CD20 between HCC and adjacent tumor tissues from GSE22058 dataset. (F) FCM analysis of PRP19 expression in immune cells (CD45+) and non-immune cells (CD45-). (G) The OS and DFS value of PRP19 and CD20 were investigated in TCGA-LIHC dataset. (H) The prognostic value of PRP19 and CD20 were investigated using Kaplan–Meier plots in Gao’s cohort. (I) Kaplan–Meier analysis combining PRP19 and CD20 levels suggested that patients with high expression PRP19 and low expression CD20 had the worst clinical outcome. *p<0.05, **p<0.01, ***p<0.001 by student’s t-test (B, D, E, F), Pearson correlation analysis (C), log-rank (Mantel-Cox) test (G, H, I). Abbreviations: LIHC, liver hepatocellular carcinoma.


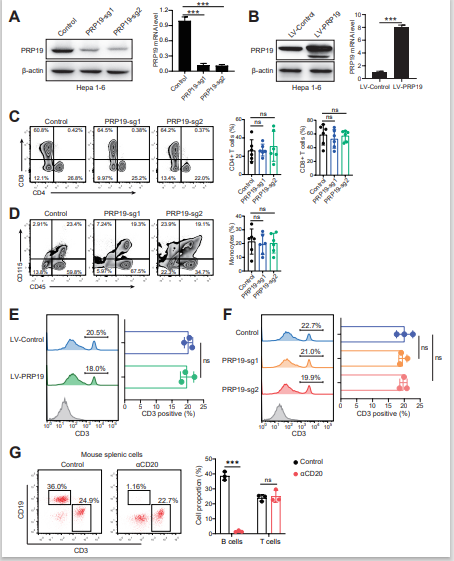


**Figure S2. Stable PRP19 knockout and overexpression HCC cell lines construction.** (A) PRP19 knockout effect in hepa1-6 cells was analyzed by western-blot and qPCR. (B) PRP19 overexpression effect in hepa1-6 cells was analyzed by western-blot and qPCR. T cells (C) and monocytes (D) proportions were analyzed by FCM in control and PRP19 knockout mice HCC tissues. CD3+ T cell migration ability was analyzed by FCM when co-cultured with PRP19 overexpression (E) and knockout HCC cells (F). (G) B cells deletion effect in mouse was analyzed by FCM. ns, not significant; ***p<0.001 by student’s t-test.


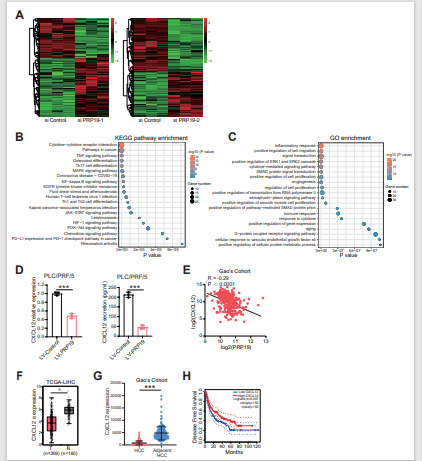


**Figure S3. RNA-sequence analysis in PRP19 knockdown HCC cells.** (A) The heatmap of differentially expressed genes. (B) KEGG pathway enrichment analysis of differentially expressed genes. (C) GO enrichment plot of differentially expressed genes. (D) CXCL12 expression was analyzed by qPCR and ELISA in PRP19 overexpression HCC cells. (E) PRP19 was negatively correlated with CXCL12 mRNA expression in Gao’s cohort. CXCL12 mRNA level was down-regulated in HCC tumor tissues from TCGA-LIHC (F) and Gao’s cohort (G). (H) Low level of CXCL12 in HCC patients indicated poor disease-free survival outcome. *p<0.05, ***p<0.001 by student’s t-test (D, F, G), Pearson correlation analysis (E) and log-rank (Mantel-Cox) test (H).


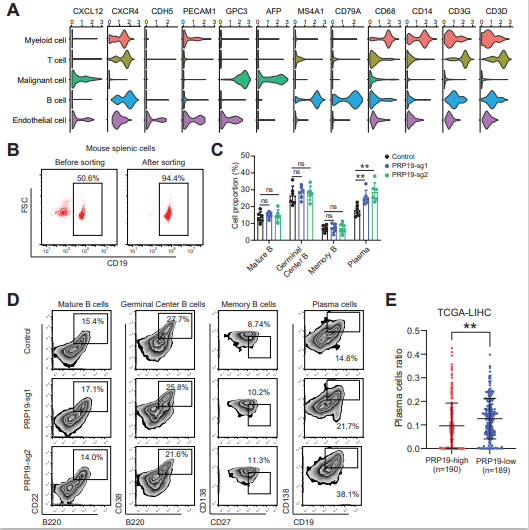


**Figure S4. PRP19 knockout HCC cells promoted plasma cell differentiation.** (A) Exploring the expression pattens of CXCL12 and CXCR4 in HCC microenvironment. (B) Mouse B cells were sorted from spleen. (C) The quantitation of mature B, germinal center B, memory B and plasma cells when co-cultured with PRP19 knockout HCC cells. (D) The proportion of mature B, germinal center B, memory B and plasma cells were analyzed by FCM when co-cultured with PRP19 knockout HCC cells. (E) Plasma cells ratio was decreased in PRP19 high expression HCC patients among TCGA-LIHC. **p<0.01 by student’s t-test (C, D). Abbreviation: ns, not significant.


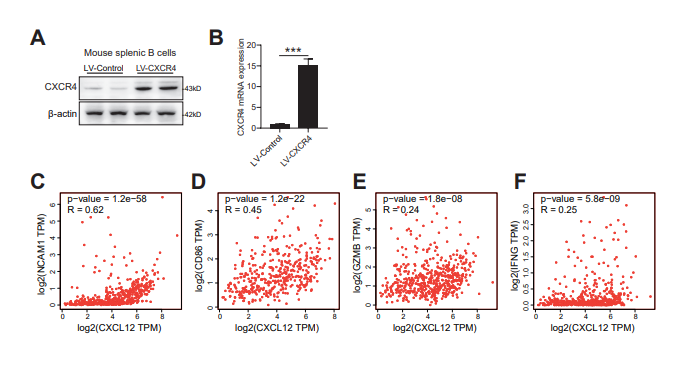


**Figure S5. Construction of CXCR4 stably expression B cells.** CXCR4 protein (A) and mRNA (B) levels were analyzed in mouse splenic B cells transfected with the lentivirus. CXCL12 was positively correlated with NCAM1 (C), CD86 (D), GZMB (E), and IFNG (F) in TCGA-LIHC dataset. *p<0.05, **p<0.01, ***p<0.001 by student’s t-test (B) and Pearson’s correlation analysis (C, D, E, F).


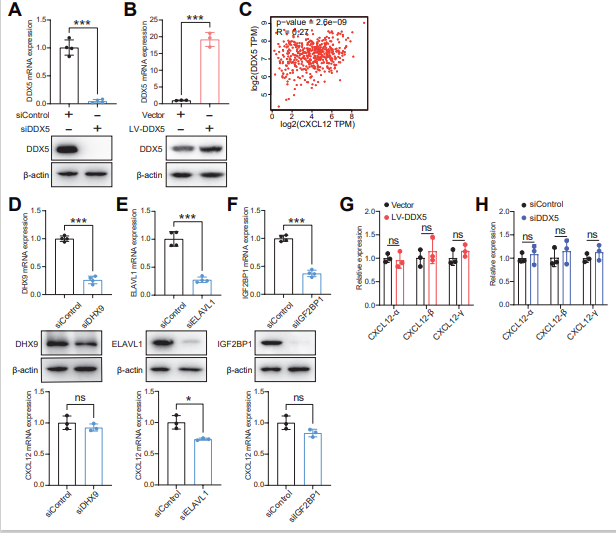


**Figure S6. DDX5 was positively correlated CXCL12 expression in HCC.** DDX5 mRNA and protein levels in DDX5 knockdown (A) and-overexpression (B) HCC cells. (C) Correlation analysis of DDX5 and CXCL12 in TCGA-LIHC dataset. (D) CXCL12 mRNA levels in DHX9 knockdown HCC cells. (E) CXCL12 mRNA level in ELAVL1 knockdown HCC cells. (F) CXCL12 mRNA level in IGF2BP1 knockdown HCC cells. Expression levels of CXCL12 splicing transcripts were analyzed by qPCR in DDX5 overexpression (G) and-knockdown (H) HCC cells. ns, not significant; *p<0.05, ***p<0.001 by Student’s t-test (A, B, D, E, F, G, H) and Pearson’s correlation analysis (C). Abbreviation: ns, not significant.


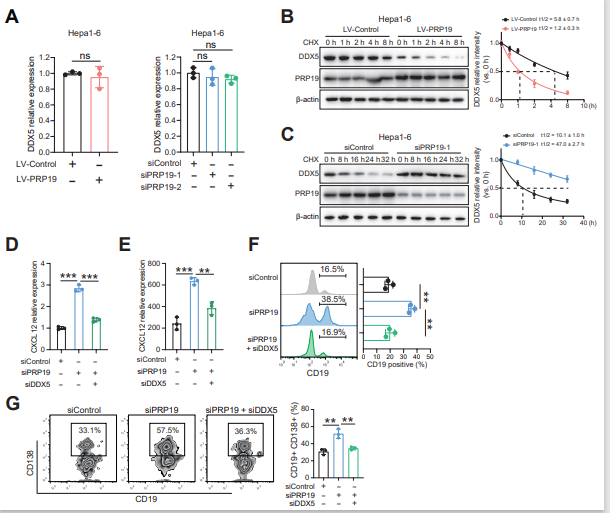


**Figure S7. PRP19 mediates DDX5 protein ubiquitination and degradation.** (A) DDX5 mRNA level was analyzed in PRP19 overexpression and knockdown mouse HCC cells by qPCR. (B) DDX5 protein degradation half time was investigated in PRP19 overexpression mouse HCC cells. (C) DDX5 protein degradation half time was investigated in PRP19 knockdown mouse HCC cells. CXCL12 mRNA (D) and protein (E) levels were analyzed in PRP19 knockdown HCC cells with or without DDX5 inhibition. (F) B cell migration was explored when co-cultured with PRP19 knockdown HCC cells with or without DDX5 inhibition. (G) Plasma cell differentiation was explored when co-cultured with PRP19 knockdown HCC cells with or without DDX5 inhibition. ns, not significant; **p<0.01, ***p<0.001 by student’s t-test (A) and ANOVA test (D, E, F, G).

**Supplementary table 1. Clinical features of 22 HCC patients for FCM analysis.**

| **Characteristics** |  | **Number** |
| --- | --- | --- |
|  |  |  |
| Age (year) | ≤50 | 8 |
|  | >50 | 14 |
| Gender | Female | 0 |
|  | Male | 22 |
| Tumor size (cm) | ≤5 | 10 |
|  | >5 | 12 |
| Tumor number | Single | 16 |
|  | Multiple | 6 |
| Tumor thrombus | No | 16 |
|  | Yes | 6 |
| Distant metastasis | No | 20 |
|  | Yes | 2 |
| Cirrhosis | No | 10 |
|  | Yes | 12 |
| TNM stage | I-II | 12 |
|  | III-IV | 10 |

**Supplementary table 2. Correlation between PRP19 expression and clinical features of HCC patient**

| **Characteristics** |  | **Tumor PRP19 expression** | | **p value** |
| --- | --- | --- | --- | --- |
|  |  | **Low (n=70)** | **High (n=50)** |  |
| Age (year) | ≤50 | 26 | 13 | 0.199 |
|  | >50 | 44 | 37 |  |
| Gender | Female | 14 | 6 | 0.246 |
|  | Male | 56 | 44 |  |
| Tumor differentiation | I-II | 29 | 20 | 0.875 |
|  | III-IV | 41 | 30 |  |
| Tumor size (cm) | ≤5 | 43 | 35 | 0.332 |
|  | >5 | 27 | 15 |  |
| Tumor number | Single | 59 | 36 | 0.102 |
|  | Multiple | 11 | 14 |  |
| Tumor thrombus | No | 32 | 27 | 0.371 |
|  | Yes | 38 | 23 |  |
| Distant metastasis | No | 57 | 39 | 0.643 |
|  | Yes | 13 | 11 |  |
| Cirrhosis | No | 45 | 23 | **0.046** |
|  | Yes | 25 | 27 |  |
| TNM stage | I-II | 28 | 18 | 0.875 |
|  | III-IV | 42 | 32 |  |
| p values were calculated using the Chi-Square test. p value of < 0.05 was considered statistically significant. | | | | |

**Supplementary table 3. List of sgRNA and siRNA sequences.**

| sgRNA | Sequence (5’-3’) |
| --- | --- |
| PRP19-sg1 (human) | GCTCATCGAGAAGTACATTG |
| PRP19-sg2 (human) | GCGCTCCGTGGTTAGCACAG |
| PRP19-sg1 (mouse) | GAGCCGGGCAATGACTCGGC |
| PRP19-sg2 (mouse) | GCGCCAGGTAGGTACCGCTC |
| siRNA | Sequence (5’-3’) |
| Control siRNA | UUCUCCGAACGUGUCACGUTT |
| PRP19-siRNA1 | GCCACUAUCAGGAUUUGGUTT |
| PRP19-siRNA2 | GCCAAGUUCAUCGCUUCAATT |
| DDX5-siRNA | GCUCUAAGUGGAUUGGAUATT |
| DHX9-siRNA | CCAACUUGAAGGAUUACUATT |
| ELAVL1-siRNA | GAGGCAAUUACCAGUUUCATT |
| IGF2BP1-siRNA | GUUCGUAUGGUUAUCAUCATT |

**Supplementary table 4. List of primers.**

| Gene name | Forward primer (5’-3’) | Reverse primer (5’-3’) |
| --- | --- | --- |
| PRP19 (human) | GCGGCTCATCGAGAAGTACA | AGCATGACTGCATCCCACTC |
| PRP19 (mouse) | CCTATCAACAACCAGCCTCTCT | GAATTCGGCCAACCCCACTC |
| CXCL12 (human) | CAGACCACGCAAGGAGTTCA | CTTCCACCTTGGAGCACTGT |
| CXCL12 (mouse) | TGACGGTAAACCAGTCAGCC | CGTGCAACAATCTGAAGGGC |
| CXCL12-α (human) | CACAGAAGGTCCTGGTGGTA | CATTGAAAAGCTGCA ATCACA |
| CXCL12-β (human) | CGCCTTTCCCAGGTGCTAAC | TGGTCTGCTTAGGGGATTTGG |
| CXCL12-γ (human) | GTGCCCTTCAGATTGTAGCC | GGGCAGCCTTTCTCT TCTTC |
| DDX5 (human) | CAAGAGCGTGACTGGGTTCT | GTGCCTGTTTTGGTACTGCG |
| DDX5 (mouse) | GCCAGTTGCTCTCAGTGGAT | AGTTCTCGAGTTGGTGCCAG |
| GZMB (mouse) | GAAGCCAGGAGATGTGTGCT | GCACGTTTGGTCTTTGGGTC |
| IFNG (mouse) | CGGCACAGTCATTGAAAGCC | TGCATCCTTTTTCGCCTTGC |
| AICDA (mouse) | AGCCATCATGACCTTCAAAGAT | GTCTGGAGAGACGAACTGAA |
| β-actin (human) | CATGTACGTTGCTATCCAGGC | CTCCTTAATGTCACGCACGAT |
| β-actin (mouse) | GGCTGTATTCCCCTCCATCG | CCAGTTGGTAACAATGCCATGT |
